# The Isolation and Replication of African Swine Fever Virus in Primary Renal-Derived Swine Macrophages

**DOI:** 10.3389/fvets.2021.645456

**Published:** 2021-03-19

**Authors:** Taehwan Oh, Duy Tien Do, Hung Van Vo, Hyeok-il Kwon, Seung-Chul Lee, Min Ho Kim, Dung Thi Thu Nguyen, Quang Tin Vinh Le, Tan Minh Tran, Toan Tat Nguyen, Joo Young Lee, Chanhee Chae

**Affiliations:** ^1^Department of Veterinary Pathology, College of Veterinary Medicine, Seoul National University, Seoul, South Korea; ^2^Faculty of Animal Sciences and Veterinary Medicine, Nong Lam University, Ho Chi Minh City, Vietnam; ^3^Department of Animal Health, Center for Veterinary Diagnostics, Regional Animal Health Office No. 6, Ho Chi Minh City, Vietnam; ^4^ChoongAng Vaccine Laboratories, Daejeon, South Korea

**Keywords:** virus replication, virus isolation, African swine fever, molecular chacterization, renal-derived macrophages

## Abstract

African swine fever virus (ASFV) causes hemorrhagic disease in domestic pigs by replicating mainly in monocyte/macrophage lineages. Various primary cells including pulmonary alveolar macrophages have been used for the propagation of ASFV on this account. However, ethical constraints and consistency problems exist as it is necessary to harvest same phenotype of primary cells in order to continue a study. We suggested renal-derived swine macrophages as a novel primary cell candidate to address these issues. These primary cells proved to be permissive to both cell adapted ASFV and a wild-type ASFV. Compared to the commercial cell line MA-104, the renal-derived macrophages were more suitable to isolate the field virus. The consistent molecular characteristics of the renal-derived macrophages were demonstrated by immunocytochemistry with antibodies against macrophage cell surface markers including CD163, CD172a, and Iba-1. Viral protein p30 and p72 expression in ASFV infected macrophages was confirmed by immunocytochemistry by use of specific monoclonal antibodies. We observed increase of cell-free viral DNA and infectious virus titer in infected cell supernatant in successive days-post-infection. These results demonstrated that primary renal-derived swine macrophages are useful for ASFV isolation and propagation in terms of cell phenotypes, susceptibility to the virus, and virus production.

## Introduction

African swine fever virus (ASFV) is an enveloped icosahedral double stranded DNA virus which belongs to the genus *Asfivirus*, family *Asfaviridae* and order *incertae sedis* (1). It causes hemorrhagic disease with high mortality rates in domestic pigs, since no successful vaccine program exists currently. Although many continuous cell lines including Vero are used for the propagation and titration of ASFV (2), the virus replicates most freely in a monocyte/macrophage lineage (3–5). The swine monocyte/macrophage lineage shows different susceptibility to ASFV due to broad phenotypes at their origins, maturation stages and activation state (6, 7). Heterogeneity of macrophage cell populations affecting permissiveness to virus infection is explained by molecular characteristics such as the virus specific membrane markers which are necessary for virus attachment and entry (8, 9).

It is well-known that ASFV replicates in various macrophage populations of spleen, lymph node, lung, liver and kidney (10–12). Among various types of macrophages assessed, pulmonary alveolar macrophages (PAMs) were suggested as they are more susceptible to ASFV infection in comparison with bone marrow derived macrophages or blood monocytes.

Maturation stage of porcine macrophages, with subsequent up-regulation of CD163 and other surface markers, is positively correlated to susceptibility to ASFV; nevertheless, CD163 is not sufficient for ASFV entry (9, 13, 14). Effect of maturation of macrophages on their susceptibility to a virus is well-studied in porcine reproductive and respiratory syndrome virus (PRRSV) where 1 day cultivated PAMs were more permissive to the virus than freshly isolated PAMs (15). Despite the many advantages of PAMs, ethical constraints exist as it is necessary to harvest large quantities in order to continue a study. In addition, it is difficult to get consistent phenotypes of macrophages between different animals. To overcome this drawback, some studies conducted propagation of ASFV using immortalized pulmonary alveolar macrophages (IPAMs), but these come with a viral titer loss in successive passages within some of the strains. Inconsistent infection kinetic studies have also supported the fact that IPAMs are not a valid candidate for ASF virus culture (5, 16–18).

Previously, a novel method of renal-derived swine macrophage culture was developed showing high harvest yields of macrophages (19). In this study, we described the replication of both a cell adapted ASFV as well as wild-type ASFV in primary renal-derived swine macrophages which were chosen due to the similar molecular characteristics that they hold when compared to PAMs.

## Materials and Methods

### Preparations of Renal-Derived Swine Macrophages

The method of isolating renal-derived macrophages from primary porcine kidney cell culture was performed as previously described with only slight modification to the methodology (19). Briefly, Primary kidney cells were harvested from 21 day-old crossbred piglets which were confirmed as free of PCV1, PCV2, PCV3, PPV, PRRSV and ASFV by PCR (20–23). RPMI-1640 culture medium containing 10% heat-inactivated fetal bovine serum (Gibco, Grand Island, NY, USA) and supplemented with 0.005 mM 2-mercaptoethanol (Sigma-Aldrich, St. Louis, MO, USA), 10 μg/mL insulin (Sigma), 100 mg/mL streptomycin (Gibco), 100 U/mL penicillin (Gibco), and 0.25 μg/mL Amphotericin B (Gibco) was used to propagate cells. After 2–3 weeks, macrophage-like round cells proliferated on a mixed monolayer cell sheet comprised of islands of polygonal and strands of spindle cells. The proliferating macrophage-like cells were easily detached from the cell sheet through gentle shaking of the culture flasks at the time of harvest. Approximately 1 × 10^6^ cells/T-75 flask floating in the culture supernatant were harvested and centrifuged (2,000 rpm for 5 min) every 3–4 days for 2 months. Harvested cells were immediately frozen in liquid nitrogen for future use with DMSO (Nacalai tesque, Kyoto, Japan).

### Immunocytochemical Characterization of Renal-Derived Macrophages

Immunocytochemistry was performed in every single harvest time point with a slight modification to the procedure as described previously (19, 24). Monoclonal antibodies against macrophage cell surface marker CD163 (Bio-Rad, Kidlington, UK; 2A10/11), CD172a (Monoclonal Antibody Center, Washington State University, USA; 74-22-15) and Iba-1 (Wako Pure Chemical Industries, Ltd, Osaka, Japan; 019-19741) were used for the immunocytochemical characterization of macrophages. Antibodies against epithelial cell markers cytokeratin 18 (CK18; Millipore Co., Billerica, MA, USA) and cytokeratin 19 (CK19; Progen, Heidelberg, Germany), along with mesenchymal cell marker α-smooth muscle actin (SMA; Progen) were used to confirm that cells were not contaminated with undesirable cells.

### Virus Infection

Three different batches of macrophages were either used uninfected as a negative control or were infected at an MOI of 0.3 with ASFV genotype II isolate (MW287337). The virus was isolated from spleen and lymph node of ASFV infected domestic pig in Vietnam, 2020, and was passaged at least 5 times in the MA-104 cell line. The wild-type virus (MW039157), the parent strain of virus isolate (MW287337), was obtained from spleen and lymph node homogenates of infected pigs, and was also used to infect the macrophages at an MOI of 0.3. After a 2 h adsorption period, cells were washed twice with Dulbecco's phosphate buffered saline (DPBS) and incubated at 37°C for the indicated times. Newly prepared MA-104 cells (kindly provided by ChoongAng Vaccine Laboratories, Daejeon, Republic of Korea) were also infected with the ASFV isolate and the wild-type ASFV in the same way to compare the permissiveness and replication efficiency with renal-derived macrophages. 96-well plates and T-25 flasks were used for macrophage and MA-104 cell cultures to evaluate viral replication.

### Replication Kinetics of ASFV

In order to determine the susceptibility of renal-derived macrophages and MA-104 to ASFV infection, the viral protein expression was analyzed by immunocytochemistry as previously described with only a slight modification (25). After 7days of incubation at 37°C, the infected cells described above were fixed and permeabilized in 80% acetone for 30 min at −20°C. After fixation, immunocytochemistry was performed using commercially available monoclonal antibodies specific for the viral protein P30 (Humimmu, Salem, NH; HI67) and P72 (Humimmu; HI68) for detecting early and late protein synthesis, respectively.

Detection of Viral DNA in cell culture supernatant was conducted for evaluating the virus production of the cells. Real-time polymerase chain reaction (RT-PCR) targeting p72 genes were performed with the infected macrophages culture supernatant at each time point (1, 3, 5, and 7 dpi). The forward and reverse primers and probe were 5′-CTGCTCATGGTATCAATCTTATCGA-3′ and 5′- GATACCACAAGATC(AG)GCCGT-3′ and 5′-(FAM)-CCACGGGAGGAATACCAACCCAGTG-3′-(TAMRA), respectively (23). Standard curve was constructed with the *C*_*T*_ values of 10-fold dilutions of the quantified ASFV DNA as described previously (26) and the RT-PCR results were shown as Log_10_ Genomic copies/mL.

The titration of live infectious virus in each cell culture supernatant was conducted as previously described, with only minor modification (27). Briefly, the presence of the infected cells was detected by immunocytochemistry, and the titers were calculated according to the Reed and Muench method by tissue culture infective dose. Results were shown as log_10_ TCID50/mL.

## Results

### Characterization of Renal-Derived Macrophages

**Figure 1:**
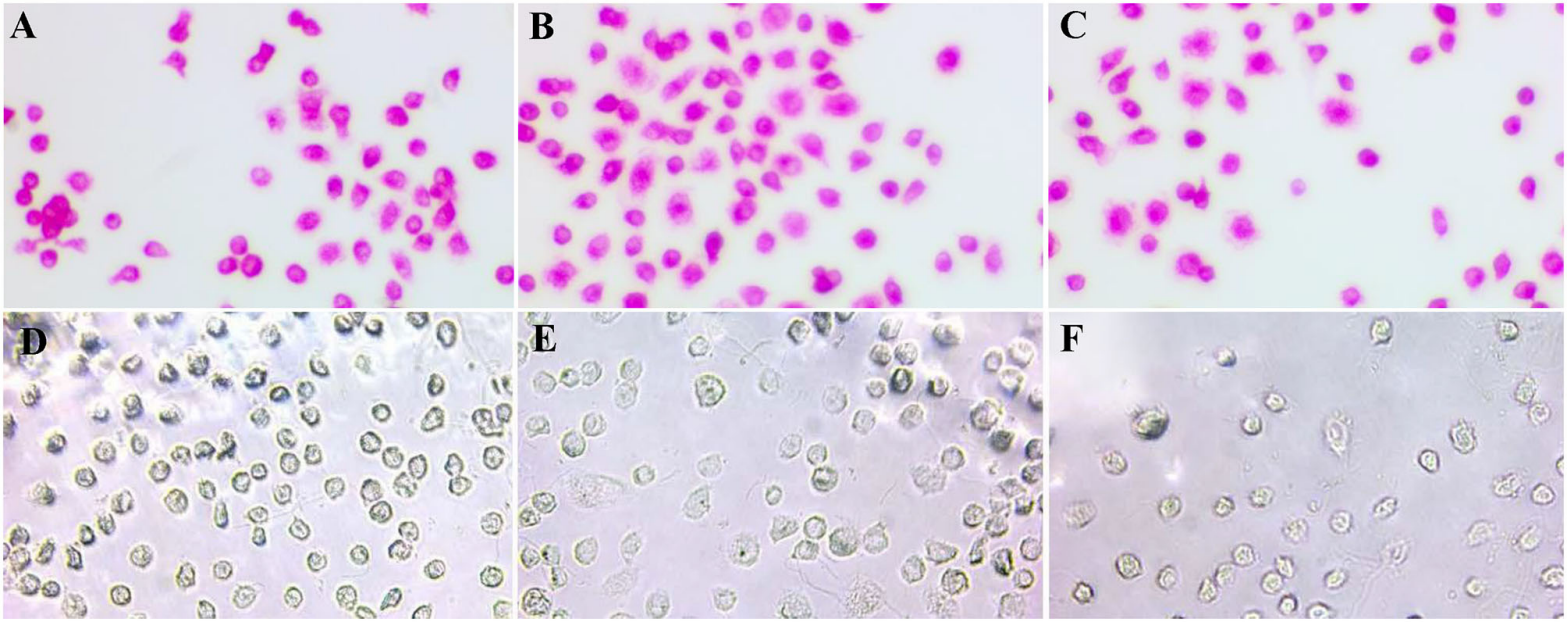
Immunocytochemical characterization of renal-derived swine macrophages using monoclonal antibodies against macrophage markers CD163 **(A)**, 172a **(B)**, and Iba1 **(C)**, epithelial cell markers CK18 **(D)** and CK19 **(E)**, and mesenchymal cell marker SMA **(F)** ×400.

Immunocytochemical characterization was performed to analyze the cell surface maker of renal-derived macrophages. Macrophage-like round cells harvested from the primary kidney monolayer cell sheet were found to be positive for macrophage markers including CD163, 172a, and Iba1, but negative for epithelial cell makers CK18 and CK19, and mesenchymal cell marker SMA in every harvest time point (Figure 1).

### ASFV Protein Expression in Renal-Derived Macrophages

**Figure 2:**
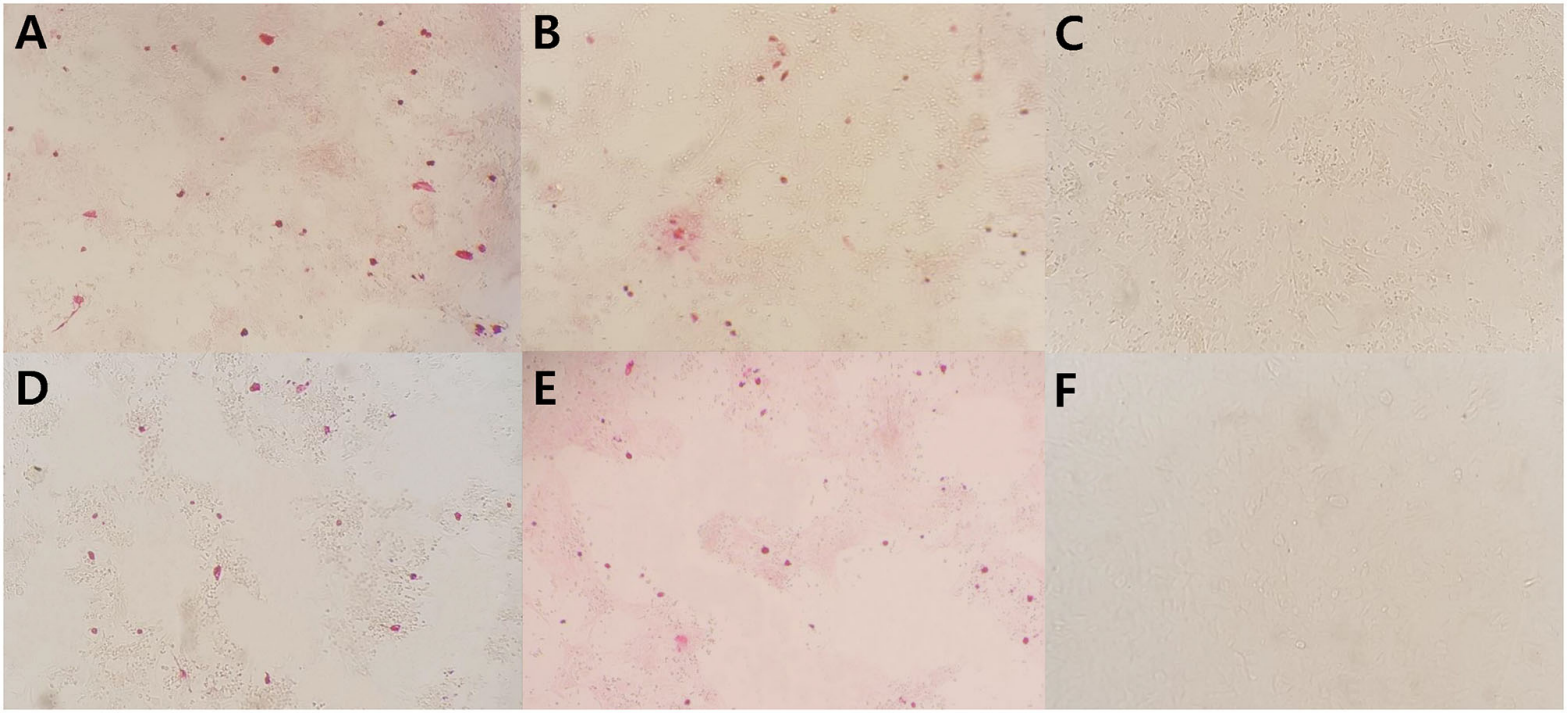
Immunocytochemistry on renal-derived macrophages infected with viral isolate strain **(A,D)**, infectious tissue homogenates **(B,E)**, and mock infected **(C,F)** using p72 and p30 antibodies, respectively, in 7 dpi. Positive signals are seen as red grains ×200.

Viral protein expression in ASFV infected renal-derived macrophages was confirmed by immunohistochemistry at 7 dpi. Both ASFV p30 and p72 antigens were detected in the cytoplasm of macrophages infected with the virus isolate or infectious tissue homogenates (Figure 2). There was no significant difference in the number of positive infected cells between the two antigens. The percentages of immunolabelled cells was ~50–60%. No signal was detected when uninfected macrophages were used as a negative control. Both macrophage monolayers infected with virus isolate and infectious tissue homogenates remained free of cytopathic effects until 7 dpi.

### ASFV Replication in Renal-Derived Macrophages and MA-104

**Figure 3:**
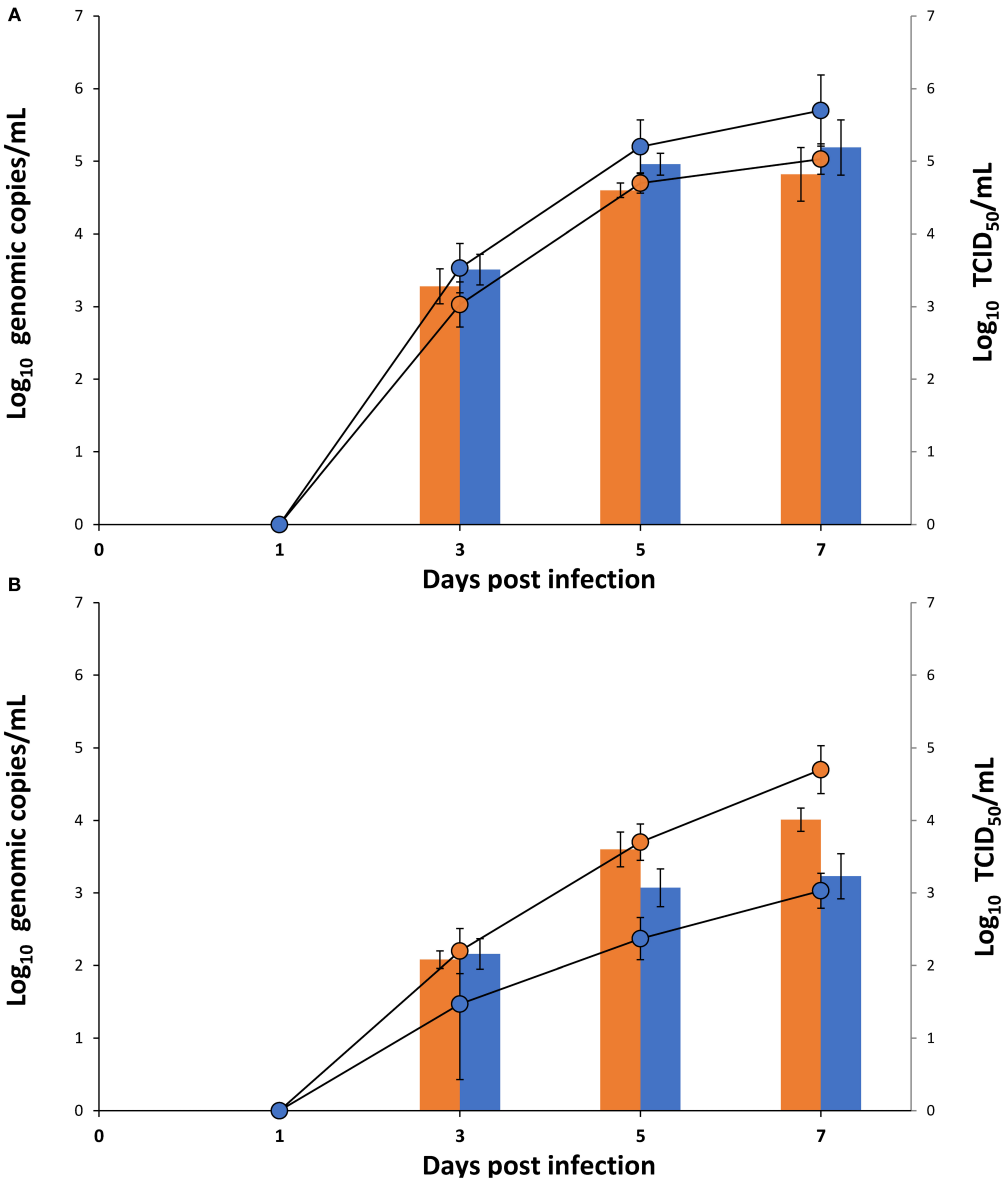
Titers of Viral DNA (bar) and infectious virus (line) of **(A)** ASFV isolate (MW287337) and **(B)** infectious wild-type virus homogenate (MW039157) were compared for renal macrophage (orange) and MA-104 (blue) cell culture supernatant at 1, 3, 5, and 7 dpi (Results were shown as Log_10_ Genomic copies/mL and log_10_ TCID_50_/mL, respectively, and each time point represents the mean of three independent experiment with different batches of macrophages and MA-104 cells).

Replication kinetics studies were designed to measure the detection of viral DNA and infectious virus in macrophage and MA-104 cell culture supernatants for the virus isolates or infectious wild-type virus homogenates at 1, 3, 5, and 7 dpi. No viral DNA and infectious virus were detected in either cell culture supernatants infected with virus isolates or infectious wild-type virus homogenate at 1 dpi, but both displayed successive increases from 3 to 7 dpi. Viral genomic copies and infectious virus titer in the MA-104 cell culture supernatant were higher than those in the macrophage cell culture supernatant at 3 to 7 dpi when (Figure 3A) for ASFV isolate (MW287337). However, for infectious wild-type virus homogenates (MW039157), viral genomic copies and infectious virus titer were similar at 3dpi for macrophage and MA-104 cell culture supernatants, but both were higher at 5 and 7 dpi for the macrophage cell culture supernatant (Figure 3B).

## Discussion

To date, the propagation of ASFV has been carried out in various types of monocyte/macrophage lineage although most of these primary cells have inevitable drawbacks including ethical constraints and poor run-to-run consistency (6–9). In this study, a novel method of ASFV cultures on primary renal-derived swine macrophages showing molecular and morphologic characteristics of monocyte/macrophage lineage was suggested with high continuity and reproducibility. Examination of cell phenotypes with immunocytochemistry revealed that these renal-derived macrophages expressed macrophage cell surface markers CD163, CD172a, and Iba-1. The majority of cells harvested for successive days in this study especially expressed a strong signal of CD163 indicating maturation of macrophages. Although expression of CD163 does not directly imply permissiveness to ASFV, it could indicate that harvested renal-derived macrophages are a mature macrophage population susceptible to the virus (9, 13, 14). Twenty, T-75 flasks containing monolayer kidney cell sheets were cultured from one, 21-days-old pig and ~1 × 10^6^ macrophage cells per T-75 flask were proliferated on a monolayer kidney cell sheet every 3–4 days for 2 months. Approximately 1 × 10^9^ of the renal macrophages were harvested, and phenotype of the cells remained consistent during this period. These results suggest ASFV propagation using primary renal-derived macrophages can reduce ethical constraint as well as consistency problem. Pulmonary alveolar macrophage, the most frequently used macrophages in ASFV studies, has proven to show highly variable function from day-to-day and between pigs (28). Even 1 day *in vitro* culture induced different results in permissiveness to PRRSV (15). It could not rule out possibility of inconsistent result from a continuing study. Primary renal-derived macrophage can be expected as a suitable alternative in that it is continuously harvested with a consistent phenotype in different batches as demonstrated in this study and the previous study (19).

In this study, both the wild-type virus and the cell adapted virus successfully replicated in renal-derived macrophages. Immunocytochemistry performed with p30 and p72 specific antibodies on fixed, infected cells, whether virus isolate or infectious tissue homogenates, resulted in some positive signal in the cytoplasm of 7 dpi cells. In ASFV-infected cells, the expression of p30 as early kinetics and p72 as late kinetics were well-studied and differentiated in time-point-based experiments as described previously (14, 29). In this day-point-based experiments, viral replication was monitored for an increase in viral titers in the cell culture supernatants, and intracellular expression of the viral proteins were only confirmed at the end of days post infection (7 dpi). An increase of the infectious virus titer was observed in 3 to 7 dpi, which correlated with an increase of virus genomic copies in infected macrophage cell culture supernatant. Previously, MA-104 was identified as a suitable commercial cell line for ASFV isolation (30), but, in this study, the renal-derived macrophages showed higher permissiveness to the wild-type virus when compared to MA-104. On this account, renal-derived swine macrophages may be suitable for the isolation of field virus and useful to understand the ASFV biology as well as diagnostic purposes. Despite the fact that renal-derived macrophages are not an immortalized cell line, overall, they can be potential alternative cell line where isolate and propagate ASFV, in particular, field virus.

Previous studies suggested that modified live ASF vaccine should be produced in primary monocyte/macrophage cells because they easily lost immunogenicity after successive culture on continuous cell lines resulting in a lack of protection against homologous virus challenge (2, 31, 32). However, the strategies for propagating modified live ASF vaccine virus in primary cells could not guarantee the supply continuity and batch-to-batch consistency. The primary renal-derived swine macrophages may be a potential alternative cell line for large-scale production of modified live ASF vaccine. Further studies are needed to confirm whether the genome and immunogenicity of the virus isolates after several passages in renal-derived macrophages is changed, in terms of the continuity and consistency.

## Data Availability Statement

The datasets presented in this study can be found in online repositories. The names of the repository/repositories and accession number(s) can be found in the article/supplementary material.

## Ethics Statement

The animal study was reviewed and approved by Seoul National University Institutional Animal Care and Use Committee.

## Author Contributions

TO and DD performance of the experimental trials, data analysis, and writing of the manuscript. H-iK, S-CL, MK, DN, QL, and TT preparation of the inoculum and lab analysis. HV, TN, and JL development of protocol. CC design of the study, review of the final manuscript, and approval for publication. All authors read and approved the final manuscript.

## Conflict of Interest

The authors declare that the research was conducted in the absence of any commercial or financial relationships that could be construed as a potential conflict of interest.
